# Nonconvulsive Status Epilepticus on Electroencephalography: An Atypical Presentation of Subacute Sclerosing Panencephalitis in Two Children

**DOI:** 10.1155/2012/374232

**Published:** 2012-10-08

**Authors:** Pratibha Singhi, Arushi Gahlot Saini, Jitendra Kumar Sahu

**Affiliations:** Department of Pediatrics, Advanced Pediatric Center, Postgraduate Institute of Medical Education and Research, Chandigarh 160012, India

## Abstract

Subacute sclerosing panencephalitis is a neurodegenerative disease secondary to measles infection that usually has a typical presentation with progressive myoclonia, cognitive decline, and periodic slow-wave complexes on electroencephalography. We report two pediatric cases who presented with periodic myoclonic jerks and cognitive decline. In both cases, the electroencephalogram showed continuous nonconvulsive status epilepticus activity. Both had elevated measles antibodies in cerebrospinal fluid and blood. Pediatricians need to be aware of this atypical presentation of subacute sclerosing panencephalitis.

## 1. Introduction

Subacute sclerosing panencephalitis (SSPE) is a progressive neurodegenerative disease caused by aberrant measles virus infection. Patients generally present with behavioral and cognitive changes, periodic myoclonus, and electroencephalogram typically shows periodic complexes [1, 2]. The diagnosis is confirmed by elevated measles antibodies in cerebrospinal fluid and serum. We report here two cases of SSPE with rare and atypical presentation of nonconvulsive status epilepticus (NCSE). 

## 2. Case  1 

A previously well, ten-year-old boy was admitted with progressive cognitive decline, myoclonic jerks, gait unsteadiness, and frequent falls for the past four months. There was no history of measles, fever, or trauma. He had received measles vaccination at nine months of age. On examination, he appeared confused with intermittent lapses in consciousness and periodic myoclonic jerks every 5 to 6 seconds in form of sudden flexion of the neck, trunk, and arms. He had difficulty in sitting, swallowing, drooling of saliva, slurred speech, and poor interaction with the examiner. He had generalized spasticity, hyperreflexia, and bilateral extensor plantar response. Cranial nerves, fundus, and the rest of the physical examination were normal. 

**Figure 1:**
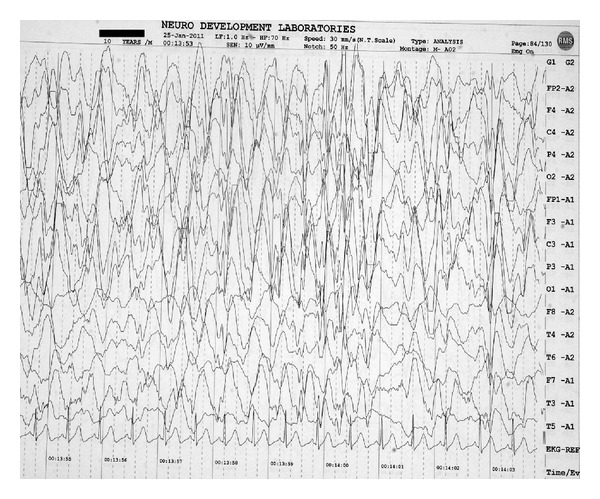
Scalp electroencephalogram showing disorganized background with generalized spike-wave discharges 2–2.5/second occupying more than 80% of tracing, consistent with nonconvulsive status epilepticus (Case  1).

**Figure 2:**
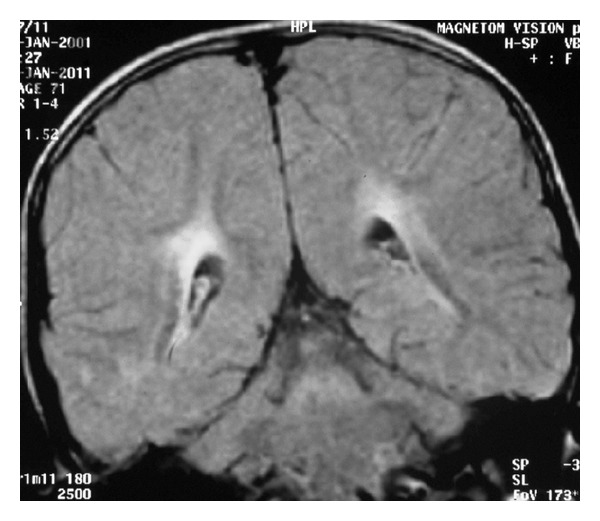
Magnetic resonance imaging of the brain (fluid attenuated inversion recovery sequence, coronal section) showing periventricular hyperintensities involving bilateral parieto-occipital white matter (Case  1).

A clinical diagnosis of SSPE was considered in view of periodic myoclonus and progressive cognitive decline. Scalp electroencephalogram showed disorganized background with generalized spike-wave discharges 2–2.5/second occupying more than 80% of tracing, consistent with NCSE (Figure 1). Following intravenous diazepam (0.3 mg/kg) administration, transient normalization of background rhythm to 6–8/second along with intermittent, generalized high-voltage slow-wave discharges was seen, but typical periodic complexes were not seen. There was no change in the mental status of the child following diazepam. Magnetic resonance imaging of brain (fluid attenuated inversion recovery sequence) showed periventricular hyperintensities involving bilateral parieto-occipital white matter (Figure 2). Measles antibody titers by enzyme-immunoassay were raised in both cerebrospinal fluid and serum: 5.24 IU/mL (positive >1.1) and 5.11 IU/mL (positive >1.1), respectively. A diagnosis of SSPE was made with clinical stage IIIA according to Jabbour's criteria [1]. Child was started on valproic acid (30 mg/kg/day) and clonazepam (0.5 mg/kg/day). At four months follow-up, the child showed some improvement in sensorium and myoclonic jerks with electroencephalogram now showing the typical periodic complexes time locked with periodic myoclonus.

## 3. Case  2 

**Figure 3:**
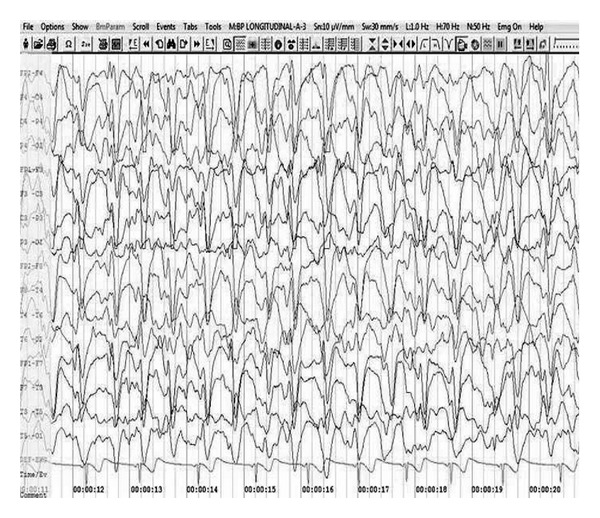
Scalp electroencephalogram showing 1-2 Hz generalized synchronous spike-wave discharges occupying more than 80% of tracing, consistent with nonconvulsive status epilepticus (Case  2).

A previously well, six-year-old boy was admitted with frequent myoclonic jerks, progressive cognitive decline, and loss of ambulation for the last one year. He had measles infection at two years of age and was not vaccinated. On examination, he demonstrated periodic jerks as sudden flexion of the neck and arms over the trunk, associated with lapse in consciousness. He had dysphagia, slurred speech, and poor interaction with the examiner. There was generalized spasticity, hyperreflexia, and bilateral extensor plantar response. Fundus examination revealed bilateral optic atrophy. Scalp electroencephalogram showed 1-2 Hz generalized, synchronous, spike-wave discharges occupying more than 80% of tracing, consistent with NCSE (Figure 3). Following intravenous diazepam (0.3 mg/kg) administration, the spike-wave discharges disappeared transiently, but periodic complexes did not occur. Also, no change in mental state was noticed. Measles antibody titers by enzyme-immunoassay were raised in both cerebrospinal fluid and serum: 1 : 625 (positive >1 : 4) and 1 : 625 (positive >1 : 256), respectively. Magnetic resonance imaging of brain (fluid attenuated inversion recovery sequence) showed periventricular hyperintensities involving predominantly occipital brain. A diagnosis of SSPE was made with clinical stage IIIA [1]. Child was started on valproic acid (30 mg/kg/day) and clonazepam (0.03 mg/kg/day). At three month follow-up, he showed some improvement in sensorium and reduction of myoclonus, but NCSE persisted on electroencephalography.

## 4. Discussion 

SSPE is one of the commonest causes of progressive myoclonia with cognitive decline in India and many other developing countries. The electroencephalogram is used as an important and quick diagnostic modality as it characteristically shows periodic complexes [2]. Shortening of the interval in periodic complexes can produce continuous slow-wave activity terminally or transient NCSE like electroencephalographic abnormalities may appear with disease progression [3]. NCSE is characterized clinically by variable amount of mental clouding, ranging from simple slowing of ideation or persistent altered sensorium to complete unconsciousness, and electrically by continuous bilateral synchronous, symmetric epileptic activity on electroencephalogram [4]. Similar to our case, Sahin et al. described NCSE in a 9-year-old boy with SSPE which improved with clonazepam [5]. Malherbe et al. observed NCSE in a child during the static period of first two years of his disease [6]. Our case underscores the importance of recognizing SSPE even in the presence of rarer, atypical electroencephalogram findings particularly in countries where the disease is endemic. 

Our first case developed SSPE despite immunization and absence of clinical measles infection. Current epidemiological and virological data suggest that measles wild-virus causes SSPE in all such cases [7]. Natural measles-virus has consistently been isolated in SSPE brain biopsy material, even in vaccinated patients with no history of natural infection [8]. As brain biopsy was not done in our patient, we presume that wild-virus infection resulted in SSPE despite immunization. Treatment with isoprinosine and interferon could not be offered in both cases due to financial constraints.

## 5. Conclusion

A high index of suspicion is needed to detect SSPE in its atypical forms, especially in measles endemic countries.
